# Molecular Cloning, Characterization, and Function of Insulin-Related Peptide 1 (IRP1) in the *Haliotis discus hanna*

**DOI:** 10.3390/genes15070960

**Published:** 2024-07-22

**Authors:** Jianfang Huang, Mingcan Zhou, Jianming Chen, Caihuan Ke

**Affiliations:** 1 Fujian Key Laboratory on Conservation and Sustainable Utilization of Marine Biodiversity, Fuzhou Institute of Oceanography, College of Geography and Oceanography, Minjiang University, Fuzhou 350108, China; jianfhuang@mju.edu.cn; 2 State Key Laboratory of Mariculture Breeding, College of Ocean and Earth Sciences, Xiamen University, Xiamen 361102, China; 22320200155965@stu.xmu.edu.cn

**Keywords:** abalone, *Haliotis discus hannai*, insulin-related peptide, growth, shellfish

## Abstract

Abalone is a popular mollusk in the marine aquaculture industry of China. However, existing challenges, like slow growth, individual miniaturization, and the absence of abundant abalone, have emerged as significant obstacles impeding its long-term progress in aquaculture. Studies have demonstrated that insulin-related peptide *(IRP*) is a crucial factor in the growth of marine organisms. However, limited studies have been conducted on *IRP* in abalone. This study indicated that the hdh-MIRP1 open reading frame (ORF) was composed of 456 base pairs, which encoded 151 amino acids. Based on the gene expression and immunofluorescence analyses, the cerebral ganglion of *Haliotis discus hannai* (*H. discus hannai*) was the primary site of hdh-MIRP1 mRNA expression. Moreover, *hdh-MIRP*1 expression was observed to be higher in the larger group than in the smaller group abalones. Only single nucleotide polymorphism (SNP) was related to their growth characteristics. However, approximately 82 proteins that may interact with *hdh-MIRP*1 were identified. The functional enrichment analysis of the 82 genes indicated that *hdh-MIRP*1 may be involved in the regulation of glucose metabolism and the process of growth. This study established a benchwork for further investigating the role of *IRP* in the growth of abalone.

## 1. Introduction

Abalone is a popular shellfish cultivated in mariculture in China. Its production is increasing annually, resulting in significant economic and social benefits. *Haliotis discus hannai* (*H. discus hannai*) is the abalone species that is most commonly cultivated in China [1]. As per the “2022 China Fishery Statistical Yearbook”, China’s aquaculture output of abalone reaches a remarkable 217,831 tons, with Fujian Province contributing approximately 80% of the entire abalone production in the country [2]. However, the challenges of slow growth and reduced size in the large-scale intensive culture have become more critical as the abalone culture industry continues to advance rapidly resulting in significant financial losses for farmers. Therefore, cultivating high-quality abalone species with fast growth is an effective way to solve this challenge. The basic idea and main focus of this study was to identify and analyze crucial molecular markers and functional genes that regulate the growth of abalone.

Recently, several studies have been carried out on the genes that regulate the growth of marine economic shellfish [3,4,5,6]. In the study of abalone, studies found that insulin-like growth factor binding protein 5 (*IGFBP5*), epidermal growth factor 1 (*EGF1*), mitogen-activated protein kinase (*MNK*), insulin receptor-related receptor (*IRR*), and fibroblast growth factor receptor (*FGFR*) were closely related to the growth of *Haliotis diversicolor* [7]. The *Haliotis diversicolor supertexta* contains SNP sites associated with growth in myostatin (*MSTN*) and bone morphogenetic protein 2 (*BMP*2) [8]. Further, *Hr-MSTN*, *Has-MIP*, *Has-SLP*, and *Has-HGAP* are linked to the growth of abalone [9,10]. Insulin-like polypeptides (ILPs) are one of the members of the insulin superfamily. Insulin-like polypeptide precursors in invertebrates are structurally similar to insulin, consisting of N-terminal signaling peptides, a B chain, a C peptide, and an A chain [11]. Studies have shown that insulin-like growth factor 1 (*IGF-*1) has a crucial role in promoting animal growth by influencing the secretion of growth hormones. It has also been observed that *IGF-*1 may efficiently induce the differentiation of muscle cells into myoduct cells, thus promoting muscle growth in *rainbow trout* [12]. The expression level of *IGF*1 mRNA in the liver and muscles of hybrid *tilapia* (*Oreochromis niloticus* ♀ × *Oreochromis aureus* ♂) with a growth advantage is higher than that of their parents, and SNPs are present at the 397th base, predominantly with allele G [13]. Similar to vertebrates, invertebrate insulin-like peptides have an important role in growth, development, reproduction, metabolism, etc. [11,14,15]. Among the shellfish, the *ILP* of *Pinctada fucata martensii* [16], *Anodonta cygnea* [17], and *Sinonovacula constricta* (*S. constricta*) [18] have all been successfully identified. The growth regulation function of *Lymnaea stagnalis* was mediated by *ILP*1, 3, 5, and 7 [15]. Several genes, like insulin-related polypeptide (*IRP*), *ILP*, *IRP*3, *IRP*3-like, and *ILP*7, have a crucial role in controlling the growth of *Crassostrea gigas* (*C. gigas*) [19,20]. Of the 31 SNPs discovered in the *ILP* of *C. gigas*, 6 showed significant associations with growth [21]. Different growth rates and expression patterns of *ILP* in *Haliotis asinina* (*H. asinina*) indicate that *ILP* is closely associated with the growth of abalone [10]. However, the physiological effects of *ILP* in *H. discus hannai* have not been investigated.

This was the first study that showed novel findings regarding the potential relationship between *IRP*1 and abalone growth. The molecular structure of *IRP*1 was examined along with the identification of SNPs that are linked to abalone growth and the analysis of its interacting protein. The results of this study will provide a deeper understanding of the molecular mechanism underlying abalone growth improvement and the inheritance of body growth in *H. discus hannai*. For the genetic improvement of abalone, it is crucial to analyze the structure and function of the main genes responsible for their growth.

## 2. Materials and Methods

### 2.1. Experimental Animals and Sample Collection

The *H. discus hannai* used in this study were obtained from Fuda Aquiculture in Jinjiang, Fujian Province, China. For the cerebral ganglion, a total of 12 larger *H. discus hannai* (“L-group”; total average weight, 8.88 ± 0.79 g; approximately 1 year old) and 12 smaller *H. discus hannai* (“S-group”; total average weight, 2.30 ± 0.42 g; approximately 1 year old) were sacrificed (*n* = 12, 12 sets of samples/group). For the examination of tissue expression, around 6 *H. discus hannai* were euthanized, and their tissues, including gonad, lymphocytes, gill, mantle, hepatopancreas, adductor muscle, cerebral ganglia, and foot tissues, were harvested for further examination (*n* = 3, 3 sets of samples/tissue). These tissues were promptly collected and preserved in liquid nitrogen, then stored at −80 °C for further analysis.

### 2.2. RNA Extraction and cDNA Preparation

Total RNA was extracted from all the respective tissues with TRIzol reagent (Invitrogen, Carlsbad, CA, USA), per the given recommendations. The product quality and purity of RNA were examined via a Nanodrop 2000 spectrophotometer (Thermo Fisher Scientific, Waltham, MA, USA). The purity and integrity of RNA samples were assessed using 1% agarose gels. Per the manufacturer’s procedure, cDNA was synthesized from RNA using a PrimerScript™ RT reagent kit with gDNA Eraser (Takara, Kusatsu, Japan).

### 2.3. Hdh-MIRP1 ORF Verification and Sequence Analysis

*H. discus hannai* genome files (provided by Dr. Caihuan Ke, Xiamen University, Xiamen) were used to design *forward*: *hdh-MIRP*1*-*F and *reverse*: *hdh-MIRP*1*-*R (Table 1), which were used to amplify the *hdh-MIRP*1 open reading frame (ORF). The sequence of predicted amino acid (aa) of the hdh-MIRP1 protein was derived using Lasergene 7.10 software. The signal peptide of the hdh-MIRP1 protein was determined using the SingalP 5.0 Server (https://services.healthtech.dtu.dk/services/SignalP-5.0, accessed on 15 July 2024) [22]. The proteolytic processing location was identified using the Prop 1.0 server (https://services.healthtech.dtu.dk/services/ProP-1.0, accessed on 15 July 2024). The secondary structure conformation of the hdh-MIRP1 protein was examined using the SOPMA Server (https://npsa.lyon.inserm.fr/cgi-bin/npsa_automat.pl?page=/NPSA/npsa_sopma.html, accessed on 15 July 2024). An analysis of the protein domains of the hdh-MIRP1 protein was carried out using the Conserved Domains Database (https://www.ncbi.nlm.nih.gov/Structure/cdd/wrpsb.cgi, accessed on 15 July 2024). Hdh-MIRP1 protein sequences were obtained from multiple species in the NCBI database and compared using the ClustalW2 tool. Next, an IRP1 phylogenetic tree was constructed using the neighbor-joining approach in the MEGA program (https://www.megasoftware.net/).

### 2.4. Expression of Recombinant Proteins, GST Pull-Down, and Identification of hdh-MIRP1-Interacting Protein

The sequence of *hdh-MIRP*1 was amplified using primers *hdh-MIRP*1-yh-F and *hdh-MIRP*1-yh-R. The resulting products were inserted into the pGEX-4T-1 vector and expressed as GST-tagged fusion protein in *E. coli* BL21 (DE3). The purified recombinant protein, GST-hdh-MIRP1, was obtained using affinity chromatography on a GST-tag purification resin (Beyotime Biotech, Shanghai, China). The purity of recombinant proteins and their expression were examined using 12% SDS-PAGE, and their detection was achieved using Coomassie blue staining [23,24]. All primers are depicted in Table 1. The total protein content of all the respective tissues of *H. discus hannai* was extracted using RIPA lysate buffer. Each tissue protein was quantified via the BCA protein quantification method [25]. First, standard samples and reaction mixture were prepared, and a standard curve was plotted based on the standard samples. After that, the samples were analyzed for tissue protein concentration [25]. These tissue proteins were mixed in equal amounts to yield 1 mL volume. This mixture of protein was added to the 50 μL resin beads with GST-hdh-MIRP1 and maintained for 2 h at 4 °C [23,24]. The control group consisted of resin beads containing only GST protein. The beads were washed with 0.01 M PBS, and the bound proteins were separated using 12% SDS-PAGE [23,25]. The gel was stained with Coomassie brilliant blue dye and destained with a decolorizing reagent [25]. The expected band was excised and then analyzed using matrix-assisted laser desorption/ionization MS/MS [23]. The MS/MS result was detected using *m*/*z* software and interpreted via MASCOT (Matrix Science) against the protein database of *H. discus hannai* [23]. Proteins with a peptide segment number ≤ 2 were eliminated, and any duplicate proteins in both the experimental (GST-HDH-MIRP1 pull-down group) and control (GST pull-down group) groups were excluded.

### 2.5. Gene Expression Profiles via RT-PCR

The gene expression of *hdh-MIRP*1 was determined using a qRT-PCR assay on a 7500FAST system (ABI, CA, USA). The relative gene expression was quantified using the 2^−∆∆CT^ method. The β-actin gene serves as an internal control gene. All specific gene primers are presented in Table 1. Approximately 20 μL of reaction mixture was prepared for PCR amplification. The mixture comprised FastStart Universal SYBR Green Master (ROX) (10 μL), primers (1 μL, 10 μM each), 100-fold dilution cDNA (5 μL), and nuclease-free water (4 μL). The cycling program was run as follows: 10 min at 95 °C, 40 cycles for 10 s at 95 °C, and 30 s at 59 °C. The intensities of all fluorescent signals were detected after the completion of the cycle.

### 2.6. Immunofluorescence

Fresh abalone head tissue was fixed in 4% paraformaldehyde for 24 h, followed by the preparation of paraffin sections. The paraffin sections were then dewaxed to water, per the routine procedure. Then sections were washed three times with PBS and blocked with 5% bovine serum albumin (BSA, Bovogen Biologicals, Keilor East, Australia) for 30 min. The paraffin sections were incubated with 50 µL of the specific primary antibody, anti-hdh-MIRP1 (GenScript, Nanjing, China), overnight at 4 °C. After reheating at room temperature for 30 min and washing thrice with PBST, the paraffin sections were incubated with the secondary antibody conjugated with FITC at 37 °C for 30 min. The cell nuclei were counterstained with 4′,6-diamidino-2-phenylindole (DAPI, Thermo Fisher Scientific, Waltham, MA, USA) for 30 min. A blocking solution containing an anti-fluorescent quencher was used for mounting. The nuclei were observed and imaged under a fluorescence microscope [26].

### 2.7. Sequence and SNP Analysis

To verify the SNPs, approximately 222 *H. discus hannai* were recruited from 10 families. A DNeasy 96 Blood and Tissue Kit (Qiagen, Shanghai, China) was used to isolate DNA content from the foot muscle samples. The phenotype was displayed by measuring the following five growth-associated characteristics: shell length and width, total weight, muscle weight, and muscle weight/wet weight ratio. The ORF sequences of hdh-MIRP1 from sampled *H. discus hannai* were genotyped by resequencing with a filter for SNPs with a minor allele frequency of less than 10%. The genome of *H. discus hannai* was used as a reference. The polymorphic information content (PIC) of the SNPs was assessed using POPGENE 1.32 software, per the provided program recommendations.

### 2.8. Statistical Analysis

Each data point was acquired from three replications. All experimental data in this study were analyzed using one-way ANOVAs in SPSS 19.0 (IBM, Tulsa, OK, USA). The significance cut-off value was considered when *p* < 0.05.

## 3. Results

### 3.1. Characterization of hdh-MIRP1 Sequence

**Figure 1:**
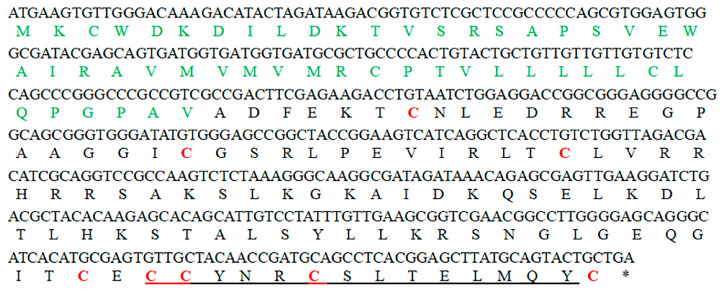
Nucleotide and aa sequences of hdh-MIRP1 from *H. discus hannai* are illustrated in Figure 1. A visual representation of the predicted signal peptide is depicted in green. Each cysteine residue is indicated in red. In the A chain, the CCX (3) CX (8) structure is indicated by an underline.

**Figure 2:**
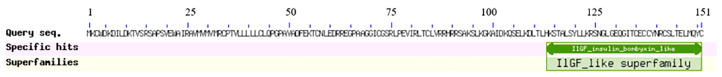
The conserved domain of hdh-MIRP1 protein in *H. discus hannai*.

**Figure 3:**
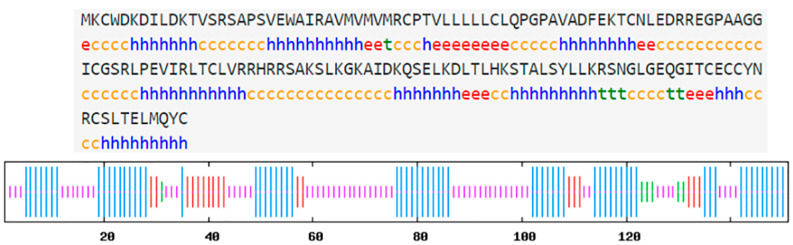
Hdh-MIRP1’s secondary amino acid structure. α-helix denoted by Hh. The extended strand is marked with Ee. β-turn is denoted by Tt. A random coil is displayed by Cc.

**Figure 4:**
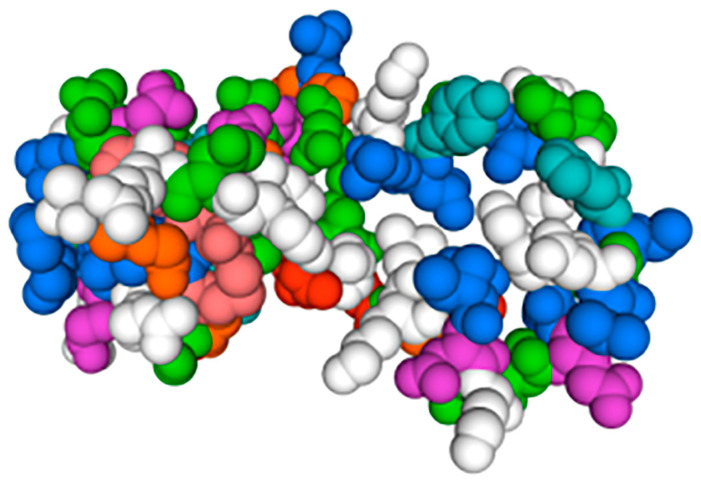
Hdh-MIRP1’s tertiary amino acid structure of hdh-MIRP1 from *H. discus hannai*.

**Figure 5:**
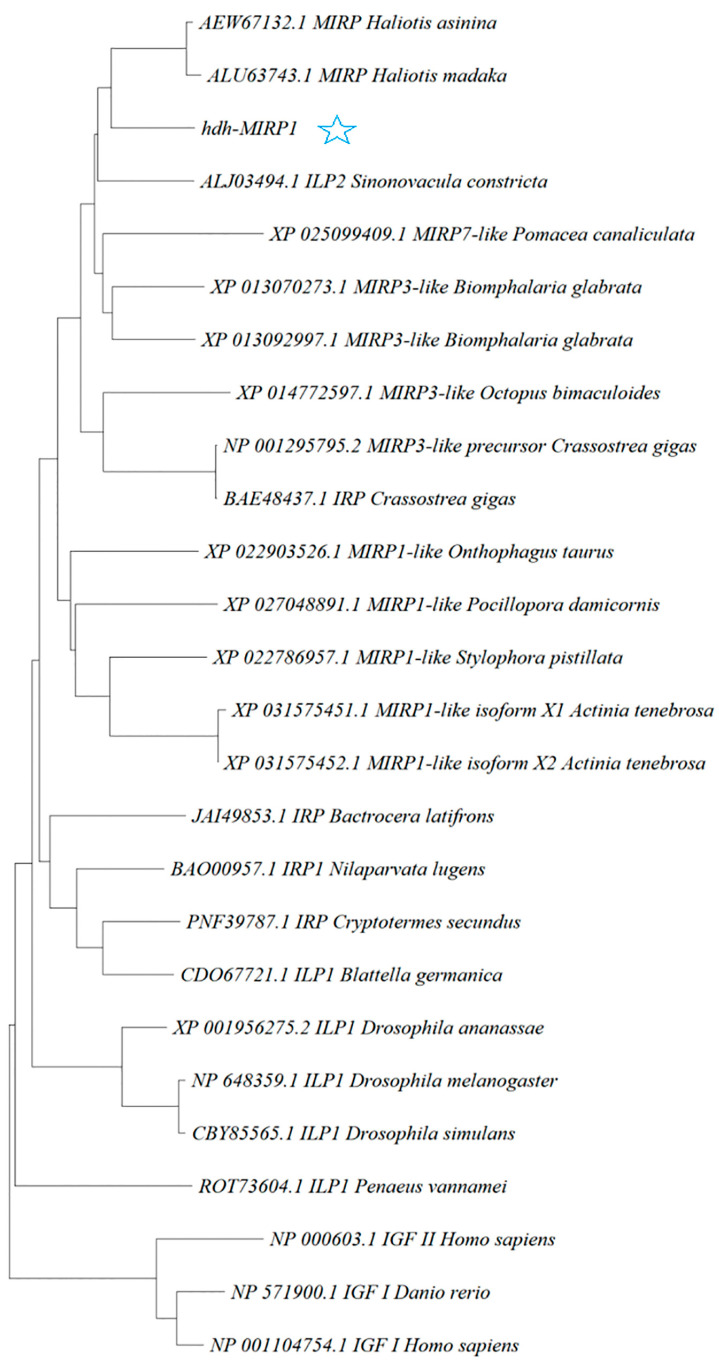
Polygenetic tree showing the relationship of the deduced *H. discus hannai* IRP1 molecule with representative IRP1 proteins of other species. A phylogenetic tree was constructed with the neighbor-joining method based on amino acid sequences using the MEGA program. The GenBank accession numbers of all sequences are shown before the name of the sequences. The pentagram represents hdh-MIRP1.

The *hdh-MIRP*1 gene consists of a full-length ORF that is 456 bp long. This ORF encodes a protein consisting of 151 amino acids. The protein sequence revealed an expected MW of 16.876 kDa and a theoretical pI of 8.672. The structure of the hdh-MIRP1 precursor (Figure 1) has similarities to those of other mollusks. The predicted aa sequence revealed a preprohormone structure characteristic of the insulin superfamily, comprising a signal peptide, a B chain, a C peptide, and an A chain. In the A and B chains of hdh-MIRP1, there were six cysteine positions frequently observed in the insulin superfamily. The presence of two additional cysteines, which have been suggested as a unique characteristic of mollusk insulins, was also confirmed in hdh-MIRP1. The cysteine in the A chain is also organized in a CCX (3) CX8). The protein domain prediction results (Figure 2) indicate that hdh-MIRP1 comprises a protein domain characteristic of the insulin-like superfamily (positions 113–151), which is classified as a member of the insulin-like superfamily. The online software of the Prop 1.0 server found that there was an RHRR (88–91 aa) protease hydrolysis site in the amino acid sequence of hdh-MIRP1. The results from the analysis of SOPMA online software indicated that the secondary structure of hdh-MIRP1 consisted of 40.40% random coils, 12.58% extended strands, and 3.97% β-turns (Figure 3). As shown in Figure 4, the protein tertiary structure of hdh-MIRP1 is similar to that of insulin. Abalones were discovered to have a close relationship via the phylogenetic analysis of IRP1 (Figure 5). The hdh-MIRP1 first clustered with *Haliotis asinina* (*H. asinina*) and *Haliotis madaka*, and then it clustered with *Sinonovacula constricta* (*S. constricta*), *Pomacea canaliculata*, and *Biomphalaria glabrata.* Thus, the structure and sequence of IRP1 proteins in shellfish appear to have undergone evolutionary conservation.

### 3.2. Expression Analysis of hdh-MIRP1

**Figure 6:**
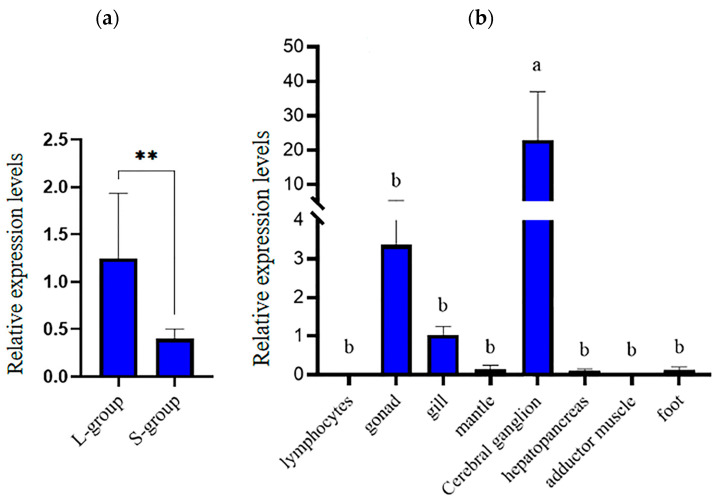
The pattern of *hdh-MIRP*1 expression in *H. discus hannai*. (**a**) Expression pattern of *hdh-MIRP*1 in the fast- and slow-growing groups. (**b**) Expression pattern of *hdh-MIRP*1 during various tissues. Three biological replicate experiments were performed for each sample. Significant differences are denoted by bars having distinct letters (*p* < 0.05), where ** are depicted as significant differences (*p* < 0.01).

**Figure 7:**
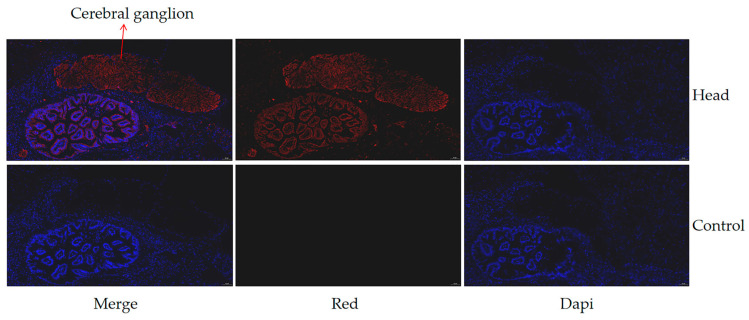
The expression of hdh-MIRP1 in the head shown using immunofluorescence. Alexa Fluor 594 (red) with DAPI nuclear counterstain (blue). Scale bar, 50 µM.

The expression of the *hdh-MIRP*1 gene in different tissues and body weights was evaluated via qRT-PCR (Figure 6). Figure 6a demonstrates that qRT-PCR analysis revealed an elevated level of *hdh-MIRP*1 mRNA in the L-group in contrast to the S-group (*p* < 0.01). The expression level of *hdh-MIRP*1 mRNA was the highest in the cerebral ganglion (*p* < 0.05) and was slightly expressed in the gonad and gill, but hardly expressed in other detected tissues of *H. discus hannai* (Figure 6b). The results of immunofluorescence show that hdh-MIRP1 was distributed in the cerebral ganglion (Figure 7).

### 3.3. Growth-Linked SNP Loci in hdh-MIRP1

A total of two SNPs were identified. The calculated average PIC value was 0.109, suggesting a relatively low level of polymorphism (PIC < 0.25). Based on the analysis, it was found that a specific SNP (the T-351C locus, Table 2) from the CDS sequence of hdh-MIRP1 showed a significant association with the growth traits of individuals from *H. discus hannai* (*p* < 0.05). At the T-351C locus, the ratio of muscle weight to total weight was substantially higher in abalones with the genotype TC compared to those with the genotypes TT and CC (*p* < 0.05).

### 3.4. Confirmation of the Interacting Proteins with the hdh-MIRP1

**Figure 8:**
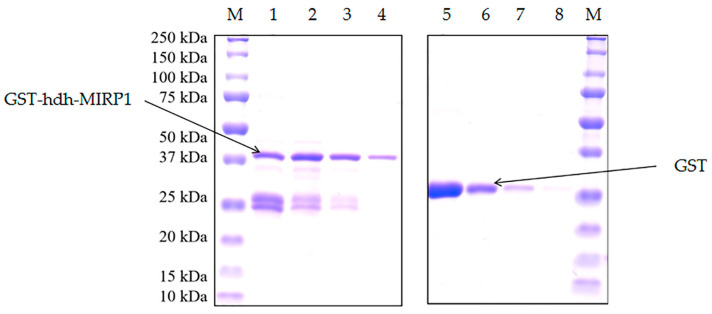
Expression of GST-hdh-MIRP1 purified recombinant protein. The “M” refers to the protein Maker (BIO-RAD 1610373). GST-hdh-MIRP1 proteins are numbered from 1 to 4. GST proteins are numbered from 5 to 8.

**Figure 9:**
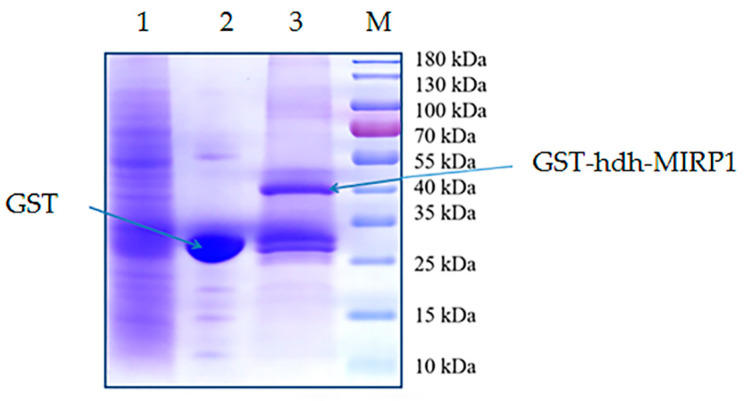
The hdh-MIRP1-interacting proteins in the adductor muscle, hepatopancreas, and cerebral ganglion of *H. discus hannai* were precipitated using GST pull-down. Lane 1 depicted the SDS-PAGE outcome corresponding to the lysis of a mixture of proteins from the adductor muscle, cerebral ganglion, and hepatopancreas of *H. discus hannai*. Lane 2 displays the bands of proteins that were extracted via mixed protein lysis combined with GST. The profiles of proteins pulled down from mixed protein lysis incubated with GST-hdh-MIRP1 were shown in lane 3. The “M” represents a protein marker (Thermofisher 26616).

**Figure 10:**
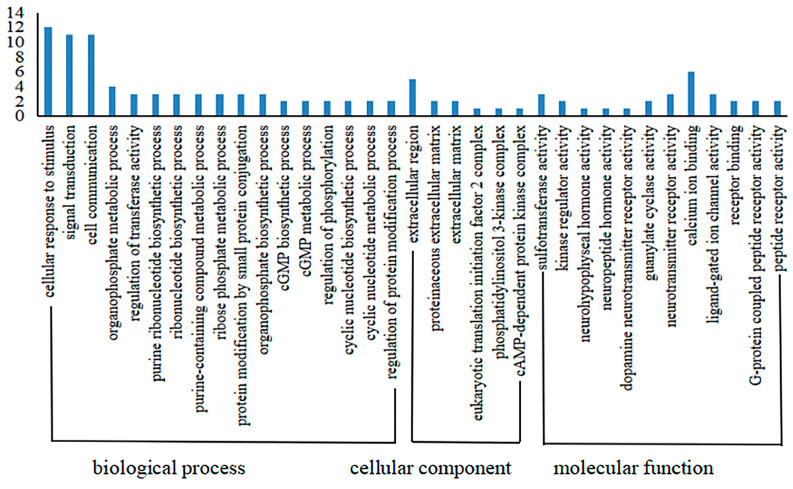
The GO annotation enrichment analysis of hdh-MIRP1-interacting proteins.

**Figure 11:**
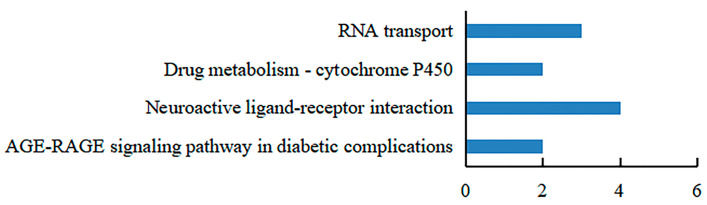
The KEGG analysis of hdh-MIRP1-interacting proteins.

Recombinant GST-hdh-MIRP1 was successfully expressed in *E. coli* BL21 (DE3) with 0.2 mM IPTG at 28 °C for 8 h. After Coomassie blue staining, the lysate of the *E. coli* BL21 expression strain reveals distinct bands at around 42 kDa on an SDS-PAGE gel; this indicates that GST-hdh-MIRP1 was expressed accurately (Figure 8). To identify the hdh-MIRP1-interacting protein, the hdh-MIRP1-interacting proteins were precipitated via the GST pull-down experiment (Figure 9). After analysis and screening, a total of 82 proteins that directly or indirectly interact with hdh-MIRP1 were identified (Table S1). Among these identified interacting proteins, neuropeptide hormone activity genes (htxA) and neurotransmitter receptor activity genes (GRHPR, α-L-fucosidase, VhaA) were found. The results of the GO enrichment analysis (Figure 10) indicate that the interacting proteins primarily have a role in various biological processes, including cellular response to stimulus, signal transduction, cell communication, etc. The cellular component mainly included the extracellular region, proteinaceous extracellular matrix, extracellular matrix, etc. The molecular function is mainly involved in the calcium ion binding, neurotransmitter receptor activity, ligand-gated ion channel activity, and so on. The KEGG analysis results (Figure 11) displayed that these interacting proteins were mainly involved in the AGE-RAGE signaling pathway in diabetic complications, neuroactive ligand–receptor interaction, drug metabolism cytochrome P450, and RNA transport.

## 4. Discussion

Insulin and its associated signaling pathway have been consistently investigated due to their crucial role in the growth and development of both vertebrates and invertebrates. The regulation of insulin superfamily members on growth and development in vertebrates has been extensively investigated. However, the current research on invertebrate shellfish is still limited. Currently, some shellfish *IRP* have been cloned, including *Lottia gigantea* [27], *L. stagnalis* [28], *S. constricta* [11], *C. gigas* [29], and *H. asinina* [10]. *H. discus hannai hdh-MIRP*1 was cloned effectively for the first time in this research. The CDS region of *hdh-MIRP*1 covered a length of 456 bp and was responsible for encoding 151 aa. The hdh-MIRP1 contained a typical insulin-like superfamily protein domain, suggesting that it belongs to the insulin-like superfamily member. Further, it comprises an N-terminal signal peptide, a B chain, a C peptide, and an A chain, all of which align with the structural characteristics in the precursors of insulin peptides in invertebrates [30,31]. It was also confirmed that hdh-MIRP1 has two more cysteines, which have been identified as a unique characteristic of mollusk insulins [29]. Moreover, the phylogenetic tree analysis demonstrated that hdh-MIRP1 was grouped with *H. asinina* and *Haliotis madaka* in a single branch, suggesting that these organisms share a highly similar genetic profile.

Recently, there has been increasing evidence that invertebrate *ILP/IRP* plays a biological function similar to that of mammalian insulin*/IGF*1. In vertebrates, *IGF*1 mainly performs the function of growth regulation and can promote animal growth [12,32]. In invertebrates, *ILP* in *Drosophila* [30] is associated with growth. Silencing *Mn-ILP* expression reduced the growth speed of *Macrobrachium nipponense* [33]. In shellfish, *ILP*1, 3, 5, and 7 of *L. stagnalis* have a role in regulating growth [15]. The growth of *C. gigas* is closely associated with *ILP* [21,29]. The *ILP* of *H. asinina* is strongly linked to growth, and its expression varies depending on the rate of abalone growth [10]. In this study, the SNP site was identified in the coding sequence of the *hdh-MIRP*1 of *H. discus hannai*, and it was found to be significantly associated with the growth characteristics. Moreover, the level of *hdh-MIRP*1 in L_group *H. discus hannai* was elevated than that in S_group abalones, suggesting a potential role for *hdh-MIRP*1 in growth regulation. The regulatory effect is positive, and this SNP site can be used as an important candidate marker for growth-associated assisted selection breeding in the future.

Tissue expression analysis also demonstrated that hdh-MIRP1 showed predominant expression in the cerebral ganglion, with slight expression in the gill, gonad, and mantle tissues and low expression in the remaining tissues. In contrast, *ILP*1 and *ILP*2 were found to be expressed in all tissue of *Sinonostricula sinonostricula* [11]. However, this finding aligns with the study outcomes of *H. asinina* [10], which primarily reveals expression in the cerebral ganglion. Further analysis depicted that *MIRP*1 expression was higher in the L-group than in the S-group abalones in *H. discus hannai*, which further proved that *MIRP*1 may actively regulate the growth of abalone. Consistent with findings from previous studies on *Aplysia californica* [34], *Lymnaeidae* [35], and *C*. *gigas* [29], this result demonstrates that *ILP* can stimulate the growth of mollusks. Further, *hdh-MIRP*1 is detected in the gonads of abalone, indicating its potential involvement in the reproductive regulation of *H. discus hannai*, along with its role in growth regulation. Related studies on the *ILP* of *C. gigas* [29] have also indicated that mollusk insulin-like peptides have this function.

Prokaryotic expression has been an effective method to study protein function in vitro. The GST pull-down test is a widely employed method in animal research for screening and validating relationships between recombinant proteins and other proteins. To better understand the process of NOS_2_ in the development of male germ cells in chickens, the interaction protein of chicken NOS_2_ was found by GST pull-down combined protein spectrometry [36]. Wang [37] demonstrated the relationship between the EcSOD protein and annexin A4 in *Nibea albiflora* using GST pull-down-combined protein spectrometry. Moreover, Xie [24] used the same approach to prove that the recombinant protein of the insulin-polypeptide receptor (ILPR) binding domain of *Sinonovacula constricta* interacts with its ILP2. Furthermore, a total of 52 proteins that directly or indirectly interact with IGF2BP in *S. constricta* have been detected using GST pull-down-combined protein spectrometry [24].

In this study, the recombinant protein GST-hdh-MIRP1 was obtained via prokaryotic expression. Protein spectrum detection technology and the GST pull-down experiment were then used to identify 82 extra proteins that potentially interact with hdh-MIRP1, either directly or indirectly. Among these candidate proteins, 12 proteins were involved in the cellular response to stimulus, suggesting that hdh-MIRP1 may be involved in the response to external stimuli. The htxA protein has the molecular function of neuropeptide hormone activity, and it is predicted that hdh-MIRP1 may cooperate with the htxA protein to regulate the life process of *H. discus hannai*. GRHPR, α-L-fucosidase, and VhaA have the function of neurotransmitter receptor activity. α-L-fucosidase belongs to the family of fucosylated oligosaccharides. This family has significant physiological functions in humans, such as growth regulation, early embryogenesis and development, and signal transduction [38]. Therefore, it is suspected that these proteins may function similarly to the hdh-MIRP1 receptor and can receive signals from hdh-MIRP1 to influence the growth of abalone.

Many studies have demonstrated that ribosomal proteins, aside from their primary function as components of ribosomes, also contribute to the regulation of cell growth and differentiation [39,40,41]. This study identified a total of 82 additional proteins, which consisted of the following 7 ribosomal proteins: RPS16, RPS24, RPS25, RPS3, RPS6, RPS9, and RPSA. Several ribosomal proteins, such as RPS6, RPL36A, and RPS15A, have been documented to enhance cell proliferation [42]. Therefore, it is predicted that hdh-MIRP1 may function in the regulation of *H. discus hannai* growth. Based on the KEGG analysis in Figure 11, these interacting proteins are significantly enriched in the AGE-RAGE signaling pathway in diabetic complications, neuroactive ligand–receptor interaction, drug metabolism cytochrome P450, and RNA transport pathways. During the commitment to cell proliferation, extensive metabolic rewiring must occur in order for cells to acquire sufficient nutrients, such as glucose, amino acids, lipids, and nucleotides, which are necessary to support cell growth [43]. Several previous studies have demonstrated the involvement of insulin and its family members in the modulation of glucose metabolism [44,45,46,47,48]. The AGE-RAGE signaling pathway in diabetic complications is involved in glucose metabolism [49]. Anabolic metabolism can provide sufficient nutrients for cell growth and proliferation [43]. These suggest that hdh-MIRP1 may regulate abalone growth by affecting nutrient supply through metabolic processes.

## 5. Conclusions

In conclusion, the CDS sequence of *hdh-MIRP*1, which was isolated from the cerebral ganglion of *H. discus hannai*, was cloned and characterized. This hdh-MIRP1 is classified as an insulin-like superfamily member and has the characteristics of an insulin-like superfamily protein domain. The cerebral ganglion of *H. discus hannai* was the primary site of *hdh-MIRP*1 expression. The level of *hdh-MIRP*1 was also higher in the L-group than in the S-group abalones. One SNP within the CDS sequence of *hdh-MIRP*1 displayed a significant correlation with growth characteristics. The protein spectrum detection technology and the results of the GST pull-down experiment revealed that 82 proteins may have interactions (direct or indirect) with hdh-MIRP1. The GO and KEGG analyses also indicated that hdh-MIRP1 may contribute to the regulation of growth, glucose metabolism, and response to external stimuli. Collectively, these results have established a benchwork for further study on the functions of insulin-like superfamily members in abalone growth.

## Figures and Tables

**Table 1:** List of primer sequences.

Primer	Sequence (5′-3′)
*hdh-MIRP*1-F	CATGAAGTGTTGGGACAAAGA
*hdh-MIRP*1-R	GATTGGTTGTGACGTCAGCAG
*hdh-MIRP*1-yh-F	CGCGGATCCATGAAGTGTTGGGACAAAG
*hdh-MIRP*1-yh-R	CCGCTCGAGGCAGTACTGCATAAGCTC
*hdh-MIRP*1-qF	AGCAGTGATGGTGATGGTGA
*hdh-MIRP*1-qR	CCGCCGGTCCTCCAGATT
*β-actin*-qF	GGTATCCTCACCCTCAAGT
*β-actin*-qR	GGGTCATCTTTTCACGGTTG

**Table 2:** Growth characteristics of *H. discus hannai* and the association between SNPs in hdh-MIRP1 (mean ± SD).

Locus	Genotype	Sample Number	Shell Length (mm)	Shell Width (mm)	Total Weight (g)	Muscle Weight (g)	Muscle Weight/Total Weight
T-213G	TT	203	73.99 ± 9.65 ^a^	49.61 ± 6.14 ^a^	41.50 ± 15.83 ^a^	16.99 ± 0.53 ^a^	0.4011 ± 0.0030 ^a^
	GT	16	71.73 ± 9.85 ^a^	50.87 ± 7.00 ^a^	42.81 ± 16.18 ^a^	18.23 ± 1.95 ^a^	0.4155 ± 0.0109 ^a^
T-351C	TT	35	72.95 ± 9.49 ^a^	49.44 ± 6.39 ^a^	40.39 ± 15.61 ^a^	16.85 ± 7.87 ^a^	0.4063 ± 0.0412 ^ab^
	TC	96	74.21 ± 9.99 ^a^	50.28 ± 6.31 ^a^	43.09 ± 16.67 ^a^	17.95 ± 7.81 ^a^	0.4086 ± 0.0428 ^a^
	CC	86	73.75 ± 9.54 ^a^	49.12 ± 6.02 ^a^	40.14 ± 14.93 ^a^	16.20 ± 7.07 ^a^	0.3957 ± 0.0411 ^b^

Note: Mean values denoted by distinct letters within a column differ substantially (*p* < 0.05).

## Data Availability

The data presented in this study are available upon request from the corresponding authors.

## Supplementary Materials

The following supporting information can be downloaded at https://www.mdpi.com/article/10.3390/genes15070960/s1: Table S1.
